# Third trimester abdominal pregnancy presenting as partial small bowel obstruction in a resource-limited setting: a case report

**DOI:** 10.1186/s13256-026-05888-1

**Published:** 2026-03-09

**Authors:** Dereje G. Andargie, Febronie Muhorakeye, Tilksew D. Abebe, Chernet T. Mengistie, Biruk T. Mengistie, Olivier N. Mbarushimana, Tite Ikuzwe, Birhanu Abera Ayana

**Affiliations:** 1 Kirehe District Hospital, Kirehe, Rwanda; 2 School of Medicine, College of Health Sciences, Addis Ababa University, Addis Ababa, Ethiopia

**Keywords:** Third-trimester abdominal pregnancy, Small bowel obstruction, Placental adhesion

## Abstract

**Background:**

Abdominal pregnancy is a rare and potentially life-threatening form of ectopic gestation in which the embryo implants within the peritoneal cavity. Diagnosis is often delayed, especially in low-resource settings, owing to limited access to early imaging and nonspecific clinical signs. While gastrointestinal symptoms may occur, intestinal obstruction is an extremely rare presentation of abdominal pregnancy.

**Case presentation:**

We report the case of an 18-year-old African Black primigravida who presented to our facility at approximately 30 weeks’ gestation with decreased fetal movements and dull abdominal pain. She had been managed at a peripheral health center 1 week earlier for presumed partial small bowel obstruction. On admission, examination revealed a fundal height larger than expected and a breech fetus with no cardiac activity on ultrasound. A visible endometrial stripe and a pelvic mass containing the fetus raised suspicion for an extrauterine pregnancy. Laparotomy confirmed a third-trimester abdominal pregnancy with intra-abdominal fetal demise. The fetus was free in the peritoneal cavity, and the placenta was attached to the omentum and small bowel. The placenta was successfully removed after careful dissection and partial omentectomy. The patient recovered well postoperatively and was discharged in stable condition.

**Conclusion:**

This case highlights the diagnostic complexity of abdominal pregnancy in low-resource environments, especially when presenting with atypical gastrointestinal symptoms. It underscores the importance of high clinical suspicion, detailed ultrasonographic assessment, and multidisciplinary preparedness in achieving favorable maternal outcomes. Early recognition and appropriate surgical intervention remain key to reducing morbidity and mortality in these rare but high-risk pregnancies.

## Background

Abdominal pregnancy (AP) is an uncommon ectopic gestation in which the embryo implants within the peritoneal cavity [1, 2]. It represents only about 1–1.5% of ectopic pregnancies, with an estimated incidence on the order of 1:10,000–1:30,000 deliveries [2, 3]. Primary AP (direct peritoneal implantation) is rare; most cases are secondary to tubal abortion or rupture [1]. Recognized risk factors include prior tubal damage or surgery, dilatation and curettage, and assisted reproduction [4]. Although very rare in high-resource regions, AP is reported more frequently in developing areas (for example, ~3.4/10,000 in Nigeria versus 1/10,000 in developed countries [3, 4]) and carries much higher maternal–fetal risk compared with normal pregnancies.

Clinically, AP often causes no specific warning signs. Typical complaints of persistent abdominal pain, nausea/vomiting, painful fetal movements, or vaginal bleeding are nonspecific [5, 6]. However, mechanical intestinal obstruction is an extremely rare manifestation. Only isolated reports in literature describe abdominal pregnancy presenting as bowel obstruction, making such presentations diagnostically challenging and easily misattributed to gastrointestinal pathology [7, 8]. Physical examination may reveal easily palpable fetal parts or an abnormal fetal lie late in pregnancy, but diagnosis ultimately relies on imaging. Unfortunately, routine ultrasound examinations often fail to recognize AP unless specifically sought. Many cases are only diagnosed during surgery or after failed induction of labor [5, 9]. A high index of suspicion plus careful ultrasonography is needed to catch AP early [10], but this is frequently not available. In low-resource settings such as ours, limited ultrasound access and trained sonographers mean extrauterine pregnancies can be missed until late in gestation [11, 12]. For example, one study reported that a pregnant woman in Niger had to travel 100 km for a scan, yet the AP was still undetected [11]. In short, without routine first-trimester imaging, AP often advances to later trimesters under the radar.

The consequences of missed AP are grave. Maternal mortality in abdominal pregnancy has been reported to be up to ~18% [13], many times higher than for normal or tubal pregnancies, and perinatal loss occurs in the majority of cases (40–95%) [2, 9]. Catastrophic hemorrhage from the placental implantation site is the chief threat, along with infection or bowel complications. Remarkably, a few cases reach term with a live neonate; even so, such infants often have deformities or complications [10, 14]. In low-resource environments, these challenges are magnified. As one report emphasizes, AP in under-resourced areas poses “significant challenges in diagnosis and management” owing to shortages of specialists and equipment [9, 11]. Improving prenatal care, training midwives, and expanding access to ultrasounds are therefore critical steps to avoid the delays and poor outcomes seen in reported cases [9].

This report highlights a rare third-trimester abdominal pregnancy that initially presented as partial small bowel obstruction, an unusual and rarely reported clinical feature [7, 8]. Unlike prior cases managed conservatively, our patient required surgical removal of a placenta adherent to both the bowel and omentum. These features, combined with the challenges of a low-resource setting, make this case a unique contribution to literature.

## Case presentation

An 18-year-old African Black female patient (gravida 1, para 0) presented to our hospital at approximately 30 weeks of gestation with complaints of absent fetal movements for 2 days and dull lower abdominal pain. She had been evaluated at a peripheral health center for diffuse abdominal pain, vomiting, and constipation 1 week earlier. At that facility, she was diagnosed with partial small bowel obstruction and managed conservatively with intravenous fluids and enemas. Her symptoms partially improved, and she was discharged without further imaging.

**Fig. 1:**
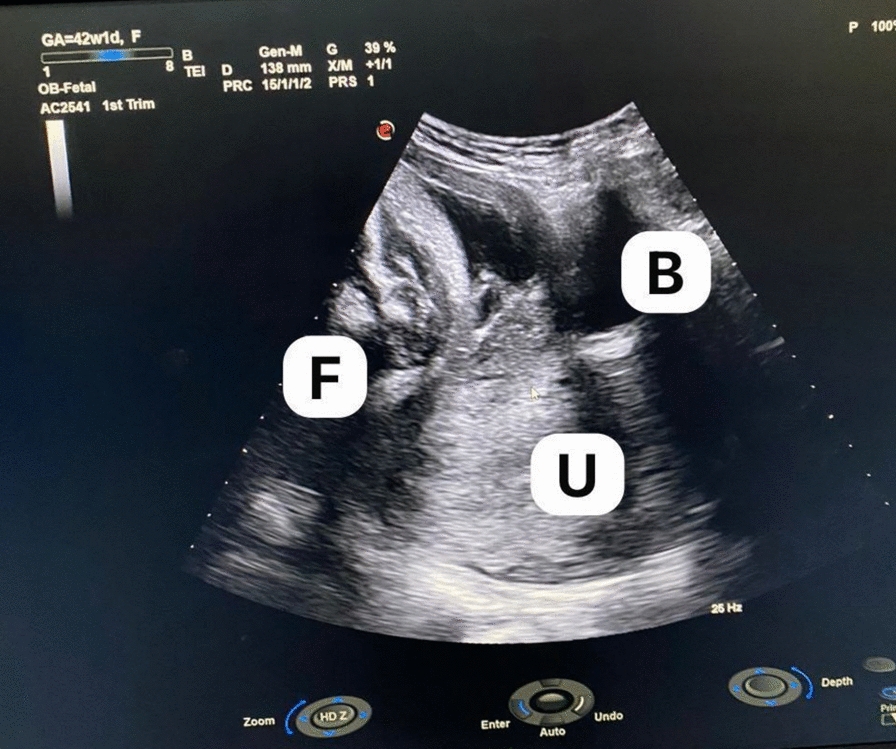
Ultrasound imaging from the initial presentation showing a breech fetus (F) that was initially misinterpreted as intrauterine. In retrospect, the fetus was located outside the uterine cavity (U). The maternal bladder (B) is also visualized

On examination, she was afebrile and hemodynamically stable. A focused obstetric exam found the uterus (or abdominal mass) to be larger than expected; symphysiofundal height measured 38 cm, suggesting growth beyond 30 weeks. On vaginal exam, the cervix was slightly open (1 cm dilated, 30% effaced) but high and posterior; the station was −3. There were no signs of labor. An ultrasound confirmed intrauterine fetal demise (IUFD), with a breech fetus misinterpreted as intrauterine (Fig. 1). The estimated weight was ~3 kg.

Given the findings of fetal demise, labor induction was initially considered. However, on re-examination the following day, a pelvic exam revealed an “intra-vaginal mass” pushing the cervix to the side, raising suspicion for extrauterine pregnancy. A repeat ultrasound showed the presence of an “endometrial stripe” separate from a large pelvic mass containing the fetus. This confirmed that the uterus might actually be empty (endometrial lining visible) and the pregnancy could be extrauterine.

Informed consent was obtained for an exploratory laparotomy with a possible hysterectomy (if uncontrollable bleeding) or other organ resection as needed. She and her family received counseling about the critical nature of her condition, including the loss of the fetus and the potential risks to her life.

**Fig. 2:**
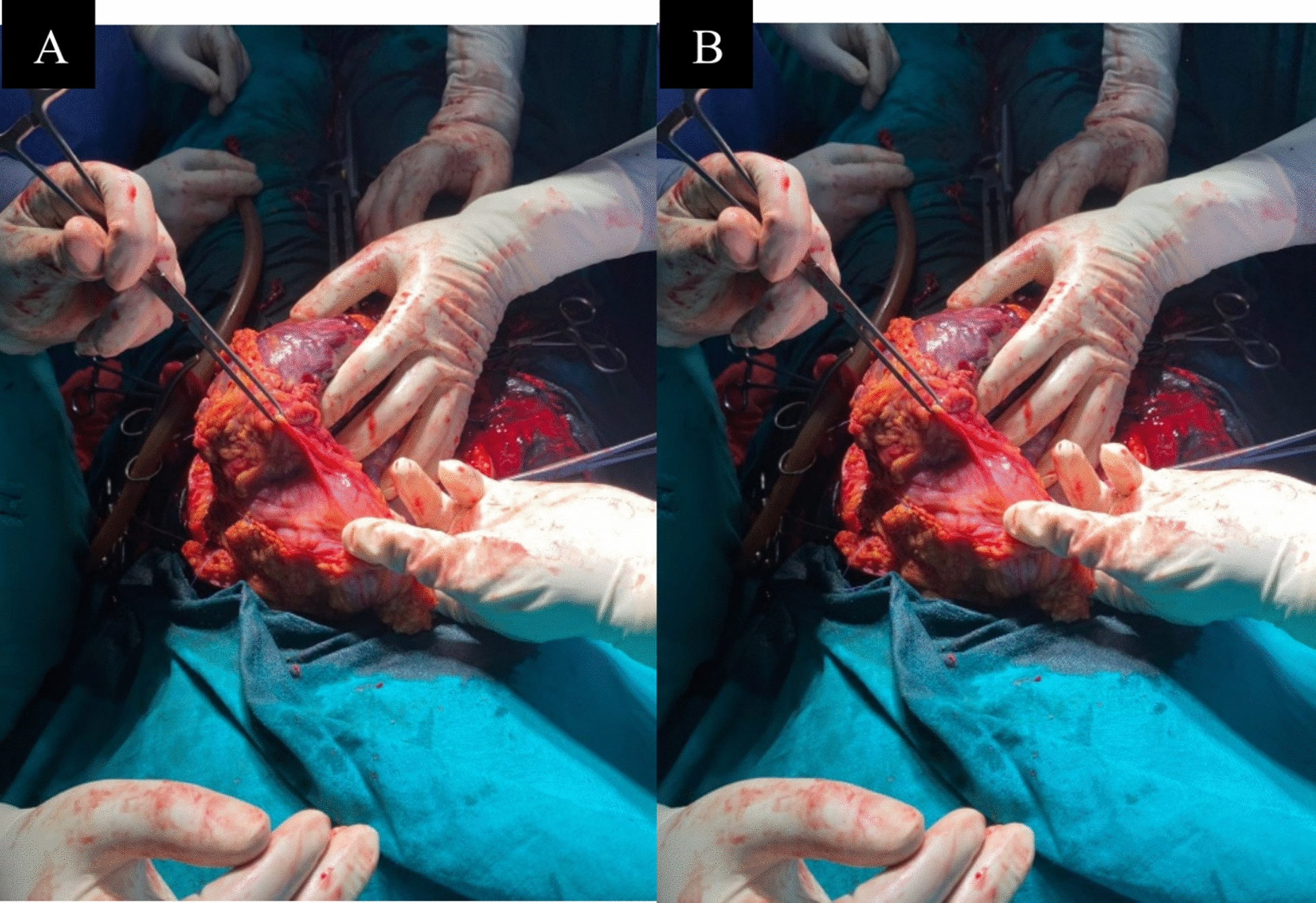
**a**, **b** Intraoperative photographs showing the fetus lying in the abdominal cavity with the placenta attached to omental and intestinal surfaces

**Fig. 3:**
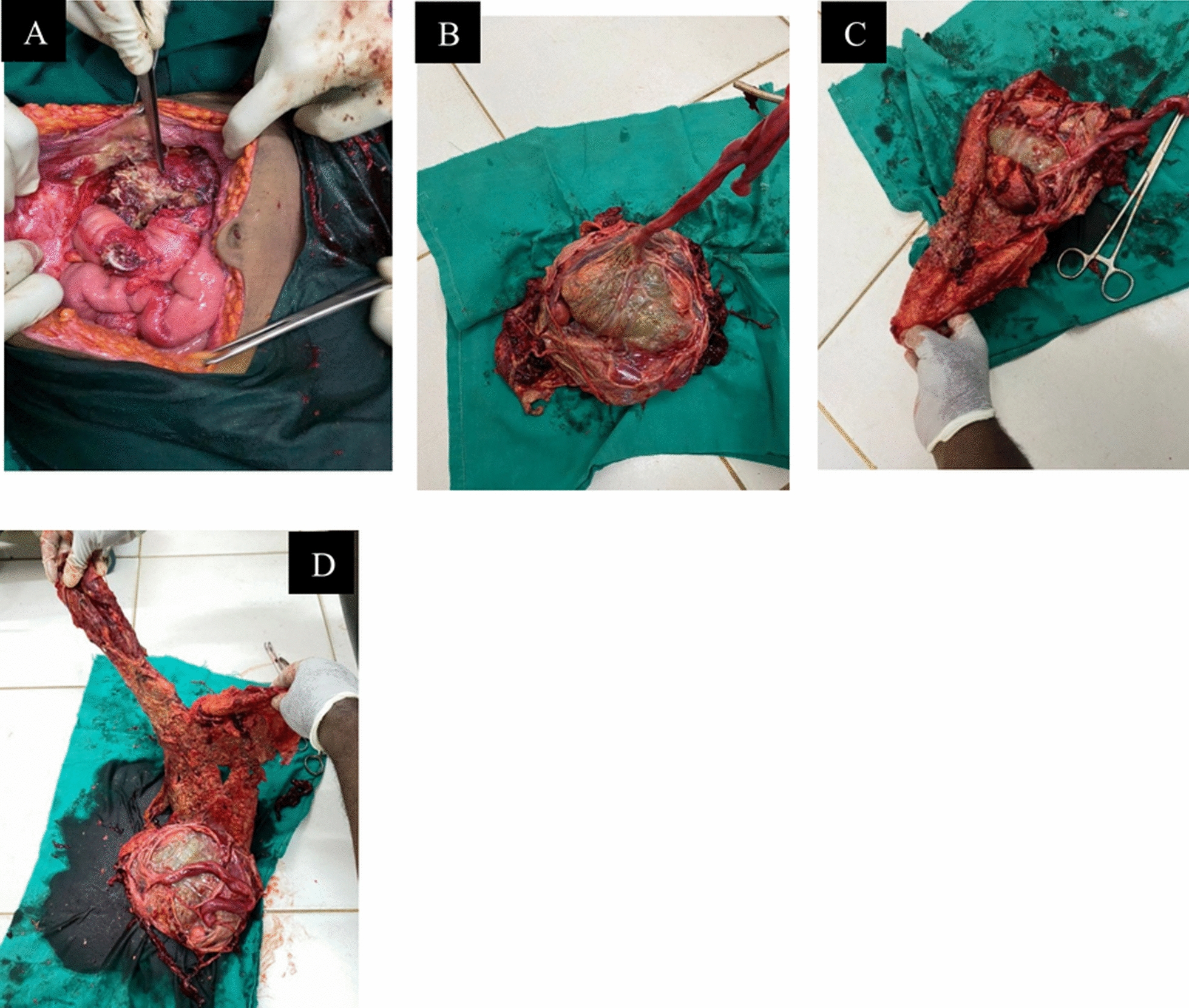
**a** Shows the operative field with the placenta attached to omental tissue; **b**, **c**, and **d** show the extracted macerated fetus

The same day, an exploratory laparotomy was performed with a midline incision extending from the pubis to just below the xiphoid to ensure adequate exposure. Upon entering the peritoneal cavity, the findings confirmed an advanced abdominal pregnancy: a well-formed fetus was found free in the abdominal cavity (Fig. 2a), enclosed by remnants of amniotic membrane and surrounded by inflammatory fluid. The placenta was attached to the omentum and loops of the small bowel, with extension to the right broad ligament/pelvic sidewall area (Fig. 2b). There was adhesion that significantly involved the small bowel with luminal narrowing in the jejunal area. The liver had soft adhesion involvement. Approximately 2.5 L of foul-smelling fluid (amniotic fluid mixed with pus and old blood) were aspirated from the abdominal cavity, reflecting the ongoing infection and hemorrhage from placental detachment. The fetus was macerated and was identified to be male, weighing ~2 kg. The fetus was carefully delivered from the abdomen (Fig. 3a, b), and the placenta was seen attached to the omentum and bowel loops (Fig. 3c, d).

The placenta was partially detached and bleeding slowly. Removal was attempted, as the attachments appeared to be mainly to the omentum and superficially to the bowel loops. Using gentle blunt dissection and cautery, a partial omentectomy was achieved to completely remove the placental tissue from the omentum and involved sections of bowel serosa. There were some areas of fibrosis (“frozen pelvis”), making dissection challenging, but no major vessels from vital organs were supplying the placenta, which facilitated removal. Bleeding was significant during placental removal, with an estimated blood loss of about 2000 mL. However, prompt clamping and ligation of bleeding omental vessels and use of electrocautery achieved hemostasis. Importantly, the uterus, ovaries, and fallopian tubes were inspected: The uterus was intact (not pregnant) and appeared to be a normal size for postpartum, confirming that the pregnancy was likely secondary abdominal (possibly from a tubal rupture earlier). The right fallopian tube or ligament area was the suspected site of origin (there was scarring in the right adnexa). No injury to the bowel was noted from the dissection, and the bowels were viable (no ischemic segment despite the prior obstruction episode). A decision was made not to leave the placenta in situ (which is sometimes done to avoid bleeding) because it had already been removed successfully, and hemostasis had been achieved. Given the blood loss, 2 units of packed red blood cells (PRBCs) were transfused intraoperatively.

The patient was discharged on the seventh postoperative day after an uneventful in-hospital course with advice to monitor for any late complications. She was also advised on family planning and the importance of early pregnancy ultrasound in the future. No significant complications were noted at her 6-week follow-up visit; she had returned to normal activities. A follow-up ultrasound showed no residual placental tissue in the abdomen.

## Discussion

Our patient’s course exemplifies the well-documented hurdles of abdominal pregnancy in resource-limited settings. Similar to the four cases recently reported from rural areas of the Democratic Republic of the Congo (DRC), our patient’s diagnosis was substantially delayed owing to constraints of local care [9]. In those reports, lack of specialists and reliance on traditional healers led to common late presentations with massive hemoperitoneum [9]. Similarly, our patient received only basic antenatal care until symptoms prompted referral. This delay mirrors the pattern found in a report in Nigeria, where 60% of AP cases were diagnosed intraoperatively owing to low suspicion and limited imaging [15]. In short, geography and healthcare infrastructure largely dictated the late stage at which the pregnancy was identified.

Even when imaging is obtained, AP can be missed. In our case, an earlier ultrasound was interpreted as a normal fetal lie, echoing other reports. Intra-abdominal pregnancies often escape detection on routine scans, reaching term undiagnosed until their characteristic findings (abnormal fetal lie, easily palpable fetal parts) trigger suspicion [16]. Specialized scanning by experienced sonographers is required to identify the empty uterus and ectopic gestation [10]. Unfortunately, neither advanced ultrasound nor magnetic resonance imaging (MRI) was available in our setting. This limitation is well recognized: A classic sonographic clue is the absence of myometrial tissue between the bladder and the pregnancy, but it was only by chance that our patient’s late scan hinted at extrauterine gestation [1, 16].

Once AP is suspected or confirmed, management must be surgical in advanced cases. As in all reviewed cases, our patient required laparotomy [9]. Intraoperatively, hemorrhage risk is the foremost concern. We prepared for transfusion and obtained blood products accordingly. In similar published cases, placental removal triggered massive bleeding. In one report, the removal of a term AP, whose placenta was on the broad ligament, required multiple blood transfusions and even factor VII to control torrential hemorrhage [14]. By contrast, some teams choose to leave the placenta in situ when removal seems too dangerous, using postoperative methotrexate to hasten involution [2]. In our patient, the placenta was partly attached to the omentum but was removed with careful ligation, in line with recommendations that optimal removal be attempted when feasible [13].

Although the fetus had demised by the time of diagnosis, the maternal course was favorable, a result that reflects the common pattern in advanced abdominal pregnancies. Literature emphasizes that advanced AP with a healthy live birth is possible but rare [10, 12]. More commonly, fetal demise or severe congenital issues occur. Even so, this successful outcome underscores that with prompt surgical intervention, even a late AP can be salvaged. Importantly, it highlights the need for preparedness: In addition to the operating team, our case demanded anesthesia support, blood availability, and postoperative intensive care—resources often scarce in rural hospitals.

Our patient ultimately had a favorable maternal outcome, though the fetus was already demised at the time of diagnosis, a result that reflects the common pattern in advanced abdominal pregnancies. Literature consistently reports that most fetuses do not survive, with perinatal mortality ranging from 40% to 95% [17]. In contrast to the rare cases where live births occur [10, 12, 16], our case is more representative of the typical course when diagnosis is delayed. Nevertheless, the maternal outcome was positive owing to prompt surgical intervention, adequate blood preparation, and careful placental management. This reinforces the importance of multidisciplinary readiness, especially in resource-limited settings, where both fetal and maternal outcomes can rapidly deteriorate if delays persist.

In summary, this report illustrates the core lessons from abdominal pregnancy reports in low-resource regions. Delayed diagnosis, driven by minimal antenatal screening and clinician suspicion, is the norm, not the exception [9]. Definitive management almost always involves laparotomy, with challenging decisions regarding the placenta and heavy transfusion requirements [4, 9, 13]. As experts have concluded, the key to improving outcomes lies in earlier recognition: enhanced prenatal screening, training of healthcare workers in AP signs, and better access to imaging in remote areas [4, 9]. Our experience reinforces that message.

## Conclusion

Abdominal pregnancy remains a rare but serious obstetric emergency, particularly when diagnosed in the third trimester. This case underscores the diagnostic challenges posed by atypical presentations such as gastrointestinal symptoms [7], which may obscure the underlying condition. In low-resource environments, where routine early ultrasound and specialist access are constrained, the likelihood of delayed recognition is high. Clinicians must therefore maintain a high index of suspicion when faced with unexplained abdominal pain, abnormal fetal lie, or discrepancies between clinical and imaging findings. Timely diagnosis and well-coordinated surgical management can significantly reduce maternal morbidity and mortality. This case reinforces the need for improved antenatal surveillance, enhanced ultrasound training, and institutional readiness to manage complex obstetric emergencies in low-resource environments.

## Data Availability

The data underlying the results presented in this work are available within the manuscript.

## Abbreviations

AP: Abdominal pregnancy
IUFD: Intrauterine fetal demise
GA: Gestational age
PRBC: Packed red blood cells
MRI: Magnetic resonance imaging
D&C: Dilatation and curettage
DRC: Democratic Republic of the Congo
